# Increased mAb production in amplified CHO cell lines is associated with increased interaction of CREB1 with transgene promoter

**DOI:** 10.1016/j.crbiot.2019.09.001

**Published:** 2019-10-05

**Authors:** Hussain Dahodwala, Prashant Kaushik, Vijay Tejwani, Chih-Chung Kuo, Patrice Menard, Michael Henry, Bjorn G. Voldborg, Nathan E. Lewis, Paula Meleady, Susan T. Sharfstein

**Affiliations:** a College of Nanoscale Science and Engineering, SUNY Polytechnic Institute, Albany, NY, USA; b National Institute for Cellular Biotechnology, Dublin City University, Dublin 9, Ireland; c Department of Bioengineering, University of California, San Diego, La Jolla, CA, USA; d Novo Nordisk Foundation Center for Biosustainability, Technical University of Denmark, 2800 Kgs. Lyngby, Denmark; e Department of Pediatrics, University of California, San Diego, La Jolla, CA, USA

**Keywords:** Chromatin immunoprecipitation (ChIP), CHO cell line selection, Nuclear proteomics, Transcriptional regulation

## Abstract

Most therapeutic monoclonal antibodies in biopharmaceutical processes are produced in Chinese hamster ovary (CHO) cells. Technological advances have rendered the selection procedure for higher producers a robust protocol. However, information on molecular mechanisms that impart the property of hyper-productivity in the final selected clones is currently lacking. In this study, an IgG-producing industrial cell line and its methotrexate (MTX)-amplified progeny cell line were analyzed using transcriptomic, proteomic, phosphoproteomic, and chromatin immunoprecipitation (ChIP) techniques. Computational prediction of transcription factor binding to the transgene cytomegalovirus (CMV) promoter by the Transcription Element Search System and upstream regulator analysis of the differential transcriptomic data suggested increased in vivo CMV promoter-cAMP response element binding protein (CREB1) interaction in the higher producing cell line. Differential nuclear proteomic analysis detected 1.3-fold less CREB1 in the nucleus of the high productivity cell line compared with the parental cell line. However, the differential abundance of multiple CREB1 phosphopeptides suggested an increase in CREB1 activity in the higher producing cell line, which was confirmed by increased association of the CMV promotor with CREB1 in the high producer cell line. Thus, we show here that the nuclear proteome and phosphoproteome have an important role in regulating final productivity of recombinant proteins from CHO cells, and that CREB1 may play a role in transcriptional enhancement. Moreover, CREB1 phosphosites may be potential targets for cell engineering for increased productivity.

## 1. Introduction

The development of hybridoma technology by Kohler and Milstein (Köhler and Milstein, 1975), set the stage for the development of monoclonal antibodies (mAbs) as tools in research, diagnostic agents, and revolutionary therapeutic agents, treating a wide range of indications. Chinese hamster ovary (CHO) cells have emerged as the dominant host for production of protein biopharmaceuticals, particularly monoclonal antibodies. As increasing numbers of therapeutic protein candidates enter various stages of development, biopharmaceutical companies are seeking innovative solutions to deliver this pipeline (Tejwani et al., 2018). Therefore, in this competitive market, it is essential to reduce time to market while maintaining desired quality attributes. Moreover, due to the large doses of antibody therapeutics required over an extended period for some indications, manufacturing capacity becomes an issue. To meet the high demand for biopharmaceuticals, many companies have built large-scale manufacturing plants containing multiple 10,000 L or larger cell-culture bioreactors. In this paradigm, high-producing cell lines significantly impact the development timelines and reduce costs by reducing needed bioreactor capacity and process cycles. Traditional cloning methods for cell line production and selection have many shortcomings and are labor intensive and time consuming. Even with the development of platform technologies and processes, each biopharmaceutical molecule still requires labor-intensive clone selection. Currently, there is a lack of understanding of the cellular organization and mechanism of high productivity, hindering the rapid development and selection of higher producing clones.

Cell line development is currently performed in the following steps:

1. A host cell line is transfected with a transgene-harboring plasmid via an optimized protocol. Flow cytometry-based staining techniques are frequently used to confirm transfected clones.
2. Pools are amplified and selected using a chemical reagent.
3. Single cells are isolated, scaled up, and adapted.
4. A final clone is selected based on titer and stability.

A top clone is not merely isolated from a pool of differential producers, rather the cell line adaptation to screening pressures results in genomic and phenotypic changes that gives rise to the final top clone (Noh et al., 2018). Methotrexate (MTX) amplification is routinely employed in dihydrofolate reductase-negative (DHFR^−^) systems to select for higher producers, with similar amplification performed using methionine sulfoximine (MSX) in glutamine synthetase deficient (GS^−^) systems. We previously characterized various CHO cell clones producing the same recombinant humanized monoclonal antibody and observed that MTX amplification leads to increased productivity by not only causing an increase in transgene copy number but also by transcriptional enhancement in higher producer CHO cell lines (Jiang et al., 2006). Thus, in these clones, the process of transcription is the rate-limiting step in recombinant antibody production. Further work demonstrated that treatment with sodium butyrate can improve gene expression in these clones (Jiang and Sharfstein, 2008). Sodium butyrate is a known histone deacetylase inhibitor (Yin et al., 2018) and improved productivity may occur by increasing the accessibility of transgene to the transcriptional machinery. Therefore, productivity can potentially be improved by altering the DNA-protein interactions in the cells.

The molecular basis for maximal expression from a defined section of DNA is dependent on the state of the chromatin. Changes in gene expression are governed by factors outside the realm of sequence information (Dahodwala and Sharfstein, 2014). These epigenetic changes are cell-type specific (Feichtinger et al., 2016; Akopov et al., 2006). Based upon epigenetic mechanisms, many strategies have been devised both to generate stably transfected clones as well as to increase specific productivity (Dahodwala and Sharfstein, 2017). While there has been considerable success in exploiting these observations to improve specific productivity, there is no clear understanding of the role of the transcriptional proteins involved. Recent computational and experimental studies exploring the interactions of transcription factors with the cytomegalovirus (CMV) promoter in the context of transient transfection and production of the reporter proteins secreted alkaline phosphatase and green fluorescent protein identified several transcription factor regulatory elements in the CMV promoter that affected transcription, particularly the cAMP response element (CRE) and nuclear factor kappa-light-chain-enhancer of activated B cells (NFκB) as positive regulatory elements and the binding site for the zinc finger regulatory protein YY1 as a negative regulatory site (Brown et al., 2015; Brown et al., 2014).

While these previous studies provide insight into potential interactions of transcription factors with the CMV promoter in CHO cells, they do not address the potential changes in transcription factor regulation during cell line selection for stable clones or chromatin modification during stable incorporation of transgenes into the host chromosomes. For example, we previously observed variability in metabolic behavior between different clones, all from the same host cell line, producing the same recombinant product, grown in the same medium under the same culture conditions, presumably as a result of modifications that occurred during the selection process (Dahodwala et al., 2012). Whether this occurs as a result of stress from increased productivity, increasing levels of MTX or other factors remains to be elucidated. In the mutable genome of the CHO cells, the changes in chromatin and nuclear proteome resulting from such adaptations will have a profound effect on the mechanism of productivity of the derived clones. Changes in protein expression and post-translational changes such as phosphorylation lead to nuclear translocation of transcription factors and subsequent changes in DNA binding (Kaushik et al., 2018). Many cofactors themselves may exhibit histone acetylation activities, thereby modifying the chromatin accessibility and subsequent gene regulation (Zupkovitz et al., 2006). These observations indicate that transgene expression may be affected by inherent differences in levels and modifications of transcription-factor binding proteins and their subsequent interaction with the promoters in different cell lines. In this study, comparative phosphoproteomic data were gathered from a mAb-producing clone (A0) and its MTX-amplified progeny (A1), using quantitative, label-free LC-MS proteomic techniques to demonstrate the activation and increased phosphorylation of CREB1 in the amplified cell line. Further, chromatin- transcription factor interactions were investigated by comparing the parental clone and its MTX-amplified progeny via chromatin immunoprecipitation (ChIP). An increased DNA-protein interaction in the higher producing cell lines was observed. CREB1 transcription factor showed ~6-fold increased association with the cytomegalovirus (CMV) promoter in higher-producing cell lines. Together, these results indicate an increasing association of transcriptional proteins with the DNA in the higher producing clones, reinforcing the notion that epigenetics and nuclear proteome interplay is an important, but poorly understood driver of transgene expression in mammalian cells.

## 2. Methods and materials

### 2.1. Cell lines

Chinese hamster ovary cell lines that produce a recombinant monoclonal humanized IgG with different specific productivities were a generous gift from an industrial collaborator. These cell lines were developed by co-transfecting two plasmids, one containing IgG heavy chain (HC) and dihydrofolate reductase (DHFR) genes and the other containing IgG light chain (LC) and neomycin phosphotransferase (Neo) genes. Transfected cell lines were initially selected in medium containing 400 μg/mL neomycin (G418). After selection, the neomycin was removed, and all subsequent cultures were performed in the absence of neomycin. Subsequently, gene amplification was performed by stepwise selection with increasing MTX concentrations. For these studies, a low producing parental cell line A0 and its amplified high-producing progeny cell line A1 were chosen for investigation. These cell lines have been previously described (Jiang et al., 2006; Jiang and Sharfstein, 2009) After culture medium adaptation, cells were cultured in a nonproprietary, serum-free medium (Dahodwala et al., 2012) containing hydrolysate and 5 mg/L recombinant human insulin (Supplementary Table 3). Growth curves and relative specific productivities are shown in Supplementary Fig. S2.

#### 2.1.1. Cell culture conditions

For every experimental method described, triplicate batch suspension cultures of all cell lines were maintained in 125 mL Erlenmeyer flasks. For each cell line, 0.2 × 10^6^ cells were seeded into 25 mL of medium and cultured on an orbital shaker at 125 rpm, 36 °C, and 5% CO_2_. Routine subculturing was carried out for 2 passages after thawing before experiments were performed.

#### 2.1.2. Sampling

Samples were taken daily from suspension cultures to determine cell density and viability. Cell densities and viabilities were estimated by hemacytometer counts (Hausser Scientific, PA) or automated counting (BioRad TC10) after diluting 1:1 with 0.4% trypan blue solution.

Cell pellets were collected at mid-exponential stage in culture (3 days after inoculation). Cells were harvested by centrifuging the appropriate volume of culture suspension at 1200 rpm for 5 min.

#### 2.1.3. Antibody assay

Antibody titers were determined by ELISA using a Human IgG ELISA Antibody Pair Kit (Stemcell Technologies) as per the manufacturer’s instructions.

### 2.2. Antibodies

Antibodies to RNA polymerase II were provided with the ChIP IT kit (Active Motif, Carlsbad CA). Antibodies to CREB1 (39013), NFκB (40916) and Sp1 (39058) were purchased separately from Active Motif, Carlsbad, CA.

### 2.3. Prediction of transcriptional proteins interacting with CMV promoter region

The Transcription Element Search System (TESS) previously available at http://www.cbil.upenn.edu/cgi-bin/tess/tess is a database that contains the various binding consensus sequences that are recorded by experimental investigation of the transcriptional proteins (Schug, 2008). It can identify binding sites using site or consensus strings and positional weight matrices from the TRANSFAC, JASPAR, IMD, and the CBIL-GibbsMat database. By querying the database with the CMV promoter sequence, we were able to generate a probability score of CMV promoter-region interactions with all transcription factor proteins in the database.

### 2.4. RNA-seq analysis

RNA-Seq data were generated as previously reported (Chiang et al., 2019). Briefly, total RNA was isolated using a Qiagen RNeasy Plus Mini Kit as per the manufacturer’s instructions. RNA quality was verified using an Agilent Bioanalyzer prior to library preparation. Library preparation was performed with an Illumina TruSeq Stranded mRNA Library Prep Kit High Throughput (Catalog ID: RS-122–2103), according to manufacturer’s protocol. Final RNA libraries were first quantified by Qubit HS and then QC on Fragment Analyzer (from Advanced Analytical). The final pool of libraries was analyzed on the Illumina NextSeq platform with high output flow cell configuration (NextSeq® 500/550 High Output Kit v2 (300 cycles) FC-404–2004).

### 2.5. RNA-Seq data processing

The RNA libraries were mapped to the CHO genome (C_griseus_v1.0) (Lewis et al., 2013; Xu et al., 2011) using STAR aligner (v. 2.5.4b) (Dobin et al., 2013). Alignments were processed to quantify gene expression counts with HTSeq-count (v. 0.7.2) (Anders et al., 2015). Genes with very low expression (less than one count in at least two samples) or of zero variance were excluded from further downstream analysis. DESeq2 with default parameters (Love et al., 2014) was used to estimate the differential expression between the A1 and A0 samples, with a positive fold change denoting higher expression in A1. The raw sequencing files and count matrix were deposited to SRA and GEO (accession number GSE133511). To comply with intellectual property requirements, the sequencing data were processed to exclude unmapped reads. This results in <5% reduction in available reads.

### 2.6. Nuclear proteomics and phosphoproteomics

For proteomic analysis, cells from three biological replicates per condition were harvested at the mid-exponential phase of the culture. The nuclear proteomic fractions were enriched using NE-PER Nuclear and Cytoplasmic Extraction Reagents (Thermo Scientific – 78,833) as per the manufacturer’s guidelines. Protein quantification was carried out using Quick Start Bradford protein Assay (BioRad). To prepare the samples for mass spectrometry analysis, 1 mg of protein lysate from each sample was reduced by adding dithiothreitol to a final concentration of 5 mM and incubated at 56 °C for 25 min. Samples were then alkylated by adding iodoacetamide to a final concentration of 14 mM and incubated for 30 min at room temperature in the dark. Alkylated samples were then vortexed and diluted at a ratio of 1:5 in 25 mM Tris-HCl. Protein samples were subsequently digested using trypsin (MS grade, Thermo Fisher Scientific) at 1:50 enzyme: substrate ratio. After a 4 h initial incubation at 37 °C, a further addition of trypsin at 1:100 enzyme: substrate ratio was performed followed by overnight incubation. After overnight digestion, trifluoroacetic acid (TFA) was added to each sample to a final concentration of 0.4% to inactivate trypsin. Peptides from the digested protein were concentrated and desalted using Sep-Pak C-18 columns with negative pressure (Villén and Gygi, 2008). Ten percent of the eluate was aliquoted for total proteome analysis. The remaining 90% was used for phosphopeptide enrichment using Fe-NTA (IMAC) spin columns (Pierce, Thermo Fisher Scientific) as per manufacturer’s instructions. Non-enriched peptide and phosphopeptide sample concentrations were determined using a Nanodrop One (Laptech International, UK).

### 2.7. LC-MS/MS analysis

Both enriched phosphopeptide samples and peptides previously collected for total proteomic analysis were dried in a SpeedVac vacuum concentrator and resuspended in 0.1% formic acid (FA) containing 2% acetonitrile (ACN). Peptide volume equivalent to 1 μg total protein was injected by autosampler for LC-MS/MS analysis using an UltiMate 3000 nanoRSLC system (Thermo Scientific) coupled in-line with an Orbitrap Fusion Tribrid mass spectrometer (Thermo Scientific). Prior to the nanoLC separation, samples were first loaded onto the trapping column (PepMap100, C18, 300 μm × 5 mm) for 3 min at a flow rate of 25 μL/min with 2% (v/v) ACN, 0.1% (*v*/v) TFA. The trapped peptides were back-flushed onto the analytical column (Easy-Spray C18 75 μm × 250 mm, 2 μm bead diameter column) using a gradient of 98% A (0.1% (v/v) FA): 2% B (80% (v/v) ACN, 0.08% (v/v) FA) to 35% B over 120 min at a flow rate of 300 nL/min.

Data-dependent product ion mode was applied for both non-enriched and phosphopeptide-enriched MS analysis. For peptide precursor fragmentation and detection, the full MS survey scan (*m*/*z* 380–1500) was performed at a resolution of 120,000 with the automatic gain control (AGC) target set to 5 × 10^5^. Peptides with charge states between 2 and 7 were selected for MS/MS with the instrument running in top speed mode with a cycle time of 3 s. Dynamic exclusion was enabled with the repeat count set to 1, exclusion duration set to 60 s and a mass tolerance of +/− 10 ppm.

For non-enriched peptide samples, MS^2^ was performed following quadrupole isolation with HCD fragmentation using normalized collision energy of 28% in the ion trap (IT). MS^2^ spectra were acquired with a fixed first m/z of 100 and an intensity threshold of 5000. AGC was set to accumulate 1 × 10^4^ ions and the maximum injection time was 35 ms.

For phosphopeptide-enriched peptide samples, multistage activation (MSA) was performed following quadrupole isolation for CID fragmentation with the normalized collision energy set to 32%, CID activation time of 10 ms and activation Q of 0.25 in the IT. An intensity threshold of 10,000 was used. The neutral loss mass for MSA was 97.9673, AGC was set to accumulate 2 × 10^4^ ions and the maximum injection time was 90 ms.

### 2.8. Quantitative label-free LC-MS/MS analysis

Relative quantitative label-free LC-MS analysis of the total proteome and phosphoproteome fractions from the two cell lines was carried out using Progenesis QI for Proteomics (Nonlinear Dynamics, Waters) in conjunction with Proteome Discoverer 2.2 (Thermo Scientific) for protein identification utilizing Sequest HT (Eng et al., 1994) search algorithm as previously described (Henry et al., 2017). Raw files generated from the MS/MS analysis were imported into Progenesis QI, and automatic reference alignment was carried out to account for retention time variability between LC runs. Upon alignment of all runs, identified features were filtered based on ANOVA *p*-value <0.05 between experimental groups. For proteomic and phosphoproteomic analysis, a Mascot generic file (mgf) was generated from all exported MS/MS spectra and analyzed using Proteome Discoverer v.2.2 (Thermo Fisher Scientific) in conjunction with SEQUEST. Peak lists were searched against a proteogenomic draft annotation for the newly assembled Chinese hamster genome which is experimentally annotated using RNA-Seq, proteomics, and Ribo-Seq (Li et al., 2019). Database search parameters were set to allow MS1 tolerance of 10 ppm; MS^2^ mass tolerance of 0.6 Da for ion trap detection; enzyme specificity was set as trypsin with two missed cleavages allowed; carbamidomethylation of cysteine was set as a fixed modification; and phosphorylation of serine, threonine, and tyrosine and oxidation of methionine (for phosphopeptide analysis) were set as variable modifications. For phosphosite identification, the PhosphoRS algorithm (Potel et al., 2019) was run through ProteomeDiscover 2.2 using diagnostic fragment ions and analyzer-specific fragment ion tolerances, as described above. Data were filtered to a 1% false discovery rate (FDR) on PSMs using automatic decoy searching in SEQUEST and by applying a phosphosite probability score of 75% or greater for S, T or Y amino acids in PhosphoRS (Potel et al., 2019). A statistical criterion of ANOVA *p*-value ≤0.05 and fold change cut-off ≥1.5 at the protein level was applied between experimental groups. Proteins with two or more unique peptides and phosphoproteins with any unique phosphopeptides that passed these criteria were then deemed to be differentially expressed between the relevant experimental groups being analyzed.

### 2.9. Purification of the DNA-protein complex

Chromatin immunoprecipitation was performed using the ChIP-IT kit (53008 Active Motif, Carlsbad CA) according to the manufacturer’s instructions. Briefly, 4 × 10^7^ cells from each cell line were harvested at day 3 and incubated with 30 mL fresh medium containing 1.5 mL 36% formaldehyde (47630 Sigma-Aldrich, St Louis, MO) for 10 min to crosslink the DNA-associated proteins to the chromatin. The reaction was stopped by washing the cells with phosphate buffered saline (PBS) and incubating with Glycine Stop-fix solution for 10 min. A final PBS wash step was used to clean the cell pellet. A sonicator (450D Branson, Danbury CT) fitted with a microtip was employed to disrupt the cells and shear the DNA to 500–1500-base pair fragments. The sonicator settings were set in accordance with the tip manufacturer’s instructions and kept at 40% amplitude. The shearing was verified by separating the sheared DNA on a 1.8% agarose gel. In subsequent steps, the Protein-DNA complex was immunoprecipitated using antibodies to CREB1, NFκB, Sp1 or RNA polymerase II. After treatment with Proteinase K and RNase to remove cellular proteins and RNA, DNA fragments were purified by using silica spin columns provided with the kit. The final elution volume in each fraction was 100 μL. This volume was concentrated to 20 μL by using a SpeedVac DNA concentrator (BC-SDNA11 Savant, GMI Inc. Ramsey, Minnesota) to obtain an adequate concentration of DNA template for RT-qPCR. ChIP was performed on three separate dates with duplicate PCR analysis for each sample.

### 2.10. RT-qPCR

Real time quantitative PCR was performed using the Roche *LightCycler*® 480 Real-Time PCR System and the LightCycler 480 Mastermix (04707494001 Roche, Indianapolis, IN). For quantification of CMV, the probe/primers combinations were as follows: forward primer: gcagagctcgtttagtgaacc; reverse primer: gaggtcaaaacagcgtggat; Universal ProbeLibrary probe: #80 (cat.no. 04689038001, Roche, Indianapolis, IN). For quantification of glyceraldehyde 3-phosphate dehydrogenase (GAPDH), the probe/primers combinations were as follows: forward primer: cgtattggacgcctggttac; reverse primer: ggcaacaacttccactttgc; Universal ProbeLibrary probe: #8 (cat.no. 04685067001, Roche, Indianapolis, IN). Reaction conditions were set up according to the manufacturer’s instructions. Crossing points (Ct) were generated from the LightCycler Software. Relative quantification of the CMV promoter and GAPDH bound to the transcription factors was performed using the 2^delta delta Ct method (Rao et al., 2013). All samples were normalized to the respective input DNA for the ChIP reaction (e.g. A0 cell line, CMV copies in input DNA) and then to sample 3 of the A0 CREB1 precipitate for CMV or GAPDH, respectively.

## 3. Results

### 3.1. Transcription factor-binding analysis of CMV promoter

In this study, protein-DNA complex interactions were examined to understand the transcriptional enhancement in the MTX-amplified cell lines and further elucidate transcriptional regulation in high productivity clones. Transcription factors associate upstream of the gene of interest via specific binding motifs that interact with consensus sequences along the promoter region to initiate transcription.

The TESS web tool was used for predicting transcription factor binding sites in the CMV promoter sequence. The CMV promoter is a strong viral promoter system used in transgene expression. It has a high level of constitutive gene expression and is efficient in a broad range of cell types. Putative transcription factors with a high probability of binding included enhancer factor C (EF-C), methylated DNA-binding protein (MDBP), activator protein 1 (AP-1), nuclear factor kappa-light-chain-enhancer of activated B cells (NFκB), activating transcription factors (ATF), cAMP response element binding protein (CREB1), and activating protein 2 (AP-2) as shown in Table 1.

### 3.2. RNA-Seq - upstream regulator analysis

Differential gene expression analysis between the A0 and A1 cell lines was performed from the count data obtained from RNA-Seq analysis using DEseq2 package in R. 6272 genes were identified as differentially expressed between the A1 and A0 cell lines (p_adj_ < 0.05) from which 2938 genes exhibited higher expression in the higher producer progeny A1 cell line, whereas 3334 genes showed lower expression in A1 as compared to the parental A0 cell line.

**Fig. 1.**
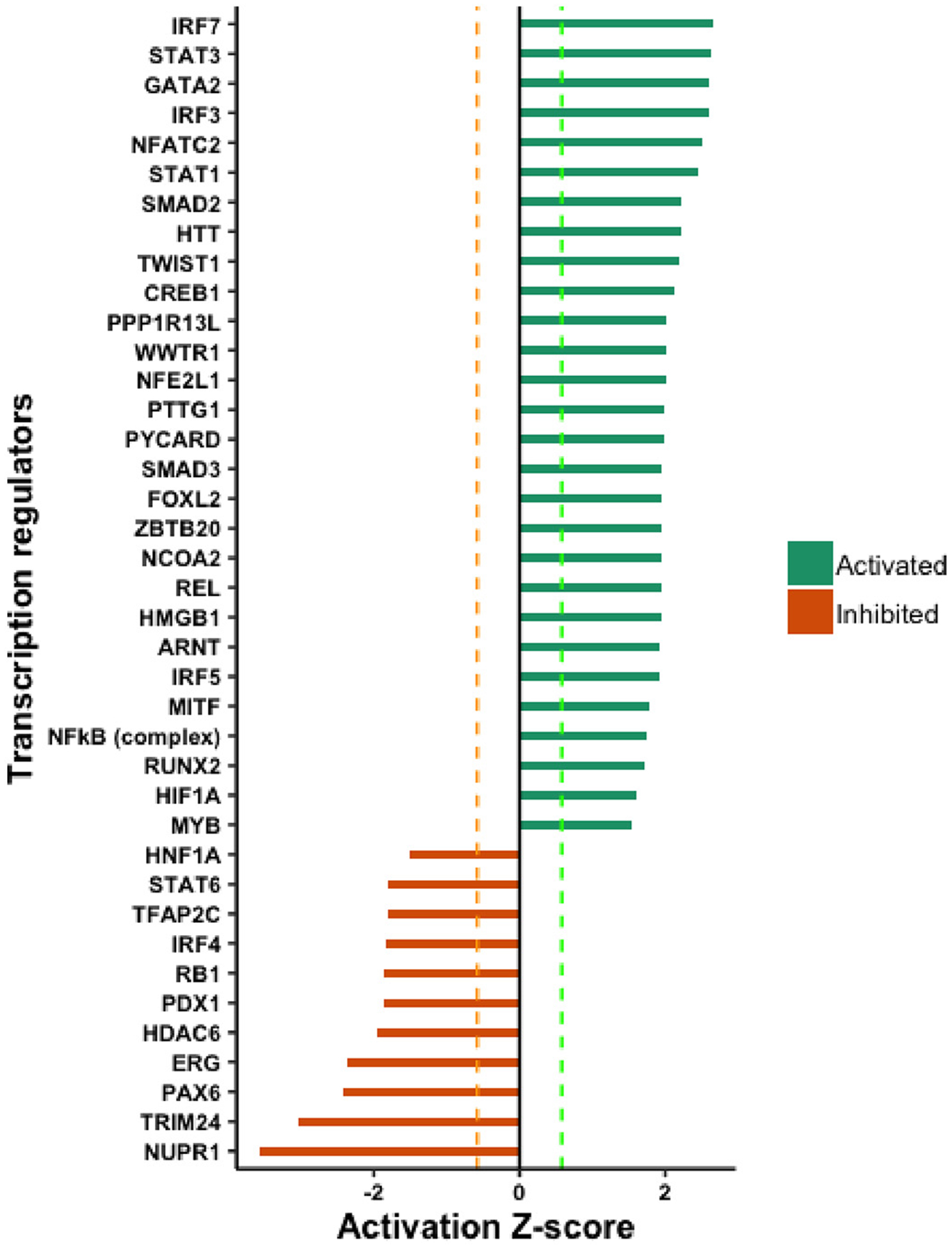
Ingenuity pathway analysis-Predicted activity status of transcription regulators from upstream pathway analysis.

To understand key genes that regulate productivity associated pathways, Ingenuity Upstream Regulator Analysis (URA) was performed on the list of differentially expressed genes. URA analysis provides a numerical account of predicted regulator activity as *Z*-score value. A positive Z-score > 1.5 indicates potential activation of a regulatory factor and Z-Score < −1.5 indicates potential inhibition of the regulatory factor (Krämer et al., 2013). We found 102 regulatory factors with Z-score > 1.5 and 56 regulatory factors with Z-score < −1.5. Among all predicted regulatory proteins, 28 transcription factors were predicted to be activated, and 11 transcription factors were predicted to be inhibited in the high producer A1 cell line. Examining the transcription factors with a high probability of interaction with the CMV promoter, both CREB1 and NFκB were identified by the URA as having a high probability of being activated in the A1 cell line compared with the A0 parental cell line. Although no expression change for CREB1 was observed at the mRNA level, URA prediction suggested strong activation of CREB1 (z-score = 2.13). In addition, the NFκB complex was identified as likely to be activated, with a z-score = 1.7. URA analysis also predicted potential activation of 11 kinases and inhibition of 6 kinases in the A1 cells, an important class of regulatory proteins that influence transcription factor activation. A complete list of all transcription factors with predicted differential activation is provided in Fig. 1.

### 3.3. Proteomic and phosphoproteomic changes associated with high productivity

Previous proteomic studies have shown that changes in protein productivity in CHO cells bring about systemic changes in the proteome (Hausmann et al., 2018) and post-translation modification of cellular proteins (Henry et al., 2017). Due to the compartmentalization of transcriptional machinery in the nucleus, studying the nuclear proteome can provide insight into changes in expression and modification of proteins that influence transgene expression. Furthermore, nuclear proteomic studies can provide up 60% greater coverage of key regulatory proteins such as transcription factors, transcription co-regulators and RNA processing proteins (Wang et al., 2017) than whole cell proteomic analysis.

Quantitative nuclear proteomic and nuclear phosphoproteomic analysis of high producer A1 cell line and low producer A0 cell line revealed 873 differentially expressed proteins (DEP) and 640 differential phosphoproteins (DEpP). From the DEP, 426 proteins were elevated in the high producer A1 cell line and 447 proteins were more highly expressed in the low producer A0 cells. Similarly, from the nuclear phosphoproteomic analysis, 528 phosphoproteins showed elevated phosphorylation and/or increased expression and 112 phosphoproteins showed reduced phosphorylation and/or decreased expression in the A1 cell line compared to the A0 parental cell line. Only proteins and phosphoproteins with fold change ≥1.5 were deemed differential. 178 proteins were common between the list of differential proteins and differential phosphoproteins. A complete list of all nuclear proteins, phosphoproteins, and phosphopeptides is presented in Supplementary Table 1.

**Fig. 2.**
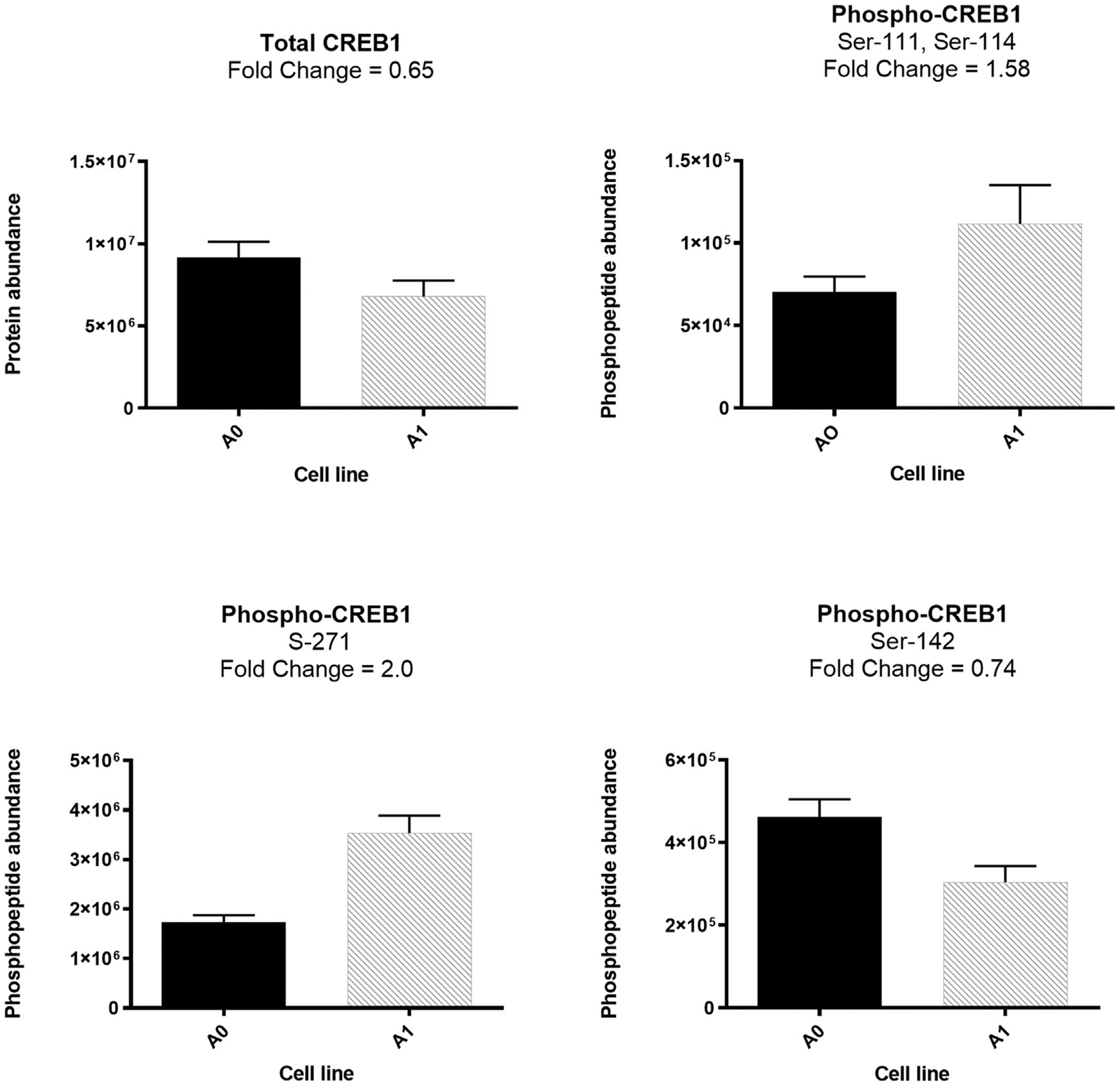
CREB1 proteomic and phosphoproteomic expression changes in the nucleus of A0 and A1 cell lines. A-Total CREB1, B-D-phosphopeptides Ser-111 and Ser-114, Ser-272, and Ser-142, respectively. Fold change is the relative abundance in A1 relative to A0.

Although no differential mRNA expression for CREB1 was identified in the transcriptomic data, 1.3-fold lower expression of total CREB1 protein was observed in the high producer A1 cell line compared with the A0 cell line (Fig. 2A, Supplementary Table 1). In the light of TESS prediction and URA analysis, the observed decrease in CREB1 total protein in the nucleus of the A1 cell line could be due to the increased activity of CREB1 in A1 cells, leading to greater CREB1-DNA association, decreasing the availability of free CREB1 in the nuclear proteome for proteomic detection. Moreover, phosphoproteomic comparison between A1 and A0 cell line revealed differential abundance of three phosphopeptides of CREB1 corresponding to four phosphosites, i.e. ILNDLSSDAPGVPR (Ser-148), RLFSGTQISTIAE SEDSQESVDSVTDSQK (Ser-111, Ser-114) and TAPTSTIAPGVVMA SSPALPTQPAEEAAR (Ser-271). The expression pattern of these phosphopeptides is shown in Fig. 2B–D (See also Supplementary Table 1).

### 3.4. CREB1: transcription regulatory relationship

The expression pattern of transcription-factor target proteins can provide insight into the dynamics of regulation by the transcription factor. Hence, CREB1-interacting proteins from TRRUST (Transcriptional Regulatory Relationships Unraveled by Sentence-based Text mining) database were searched in the lists of differential genes, proteins, and phosphoproteins between the A0 and A1 cell lines. The expression pattern of the six CREB1 target proteins identified is shown in Table 2. Two proteins that are repressed by CREB1 activation, HMOX1 and JUN, were downregulated at the mRNA and protein level, and protein JUN also showed downregulation at the phosphoprotein level in the A1 cell line. Both proteins showed a high likelihood of inhibition in the URA analysis. Two proteins that are up-regulated by CREB1, NOLC1 and NDC80, were upregulated at the total protein level in the A1 cell line. Protein NOLC1 was also upregulated at the phosphoprotein level whereas the mRNA level for NOLC1 was downregulated in the A1 cell line.

### 3.5. Chromatin immunoprecipitation

Previous work in our lab has identified transcription as the rate-limiting step in the production of monoclonal antibodies in both the parental cells and amplified progeny in these cell lines (Jiang et al., 2006). Based upon the consensus binding sequences of the transcriptional proteins and their occurrences along the CMV promoter, bioinformatic analysis of the CMV sequence indicated that CREB1 exhibits a high probability of influencing transcription from the CMV promoter (Table 1). The URA and nuclear proteomic and phosphoproteomic analysis further substantiated the potential role of CREB1. The URA also indicated a high likelihood of NFκB activation; however, we were unable to find any additional proteomic or phosphoproteomic evidence to support this activation. To evaluate whether high and low productivity clones exhibited differential interactions between the CMV promoter region and these transcription factors, chromatin immunoprecipitation was carried out, employing antibodies to these transcriptional proteins as well as to Sp1, a negative control.

**Fig. 3.**
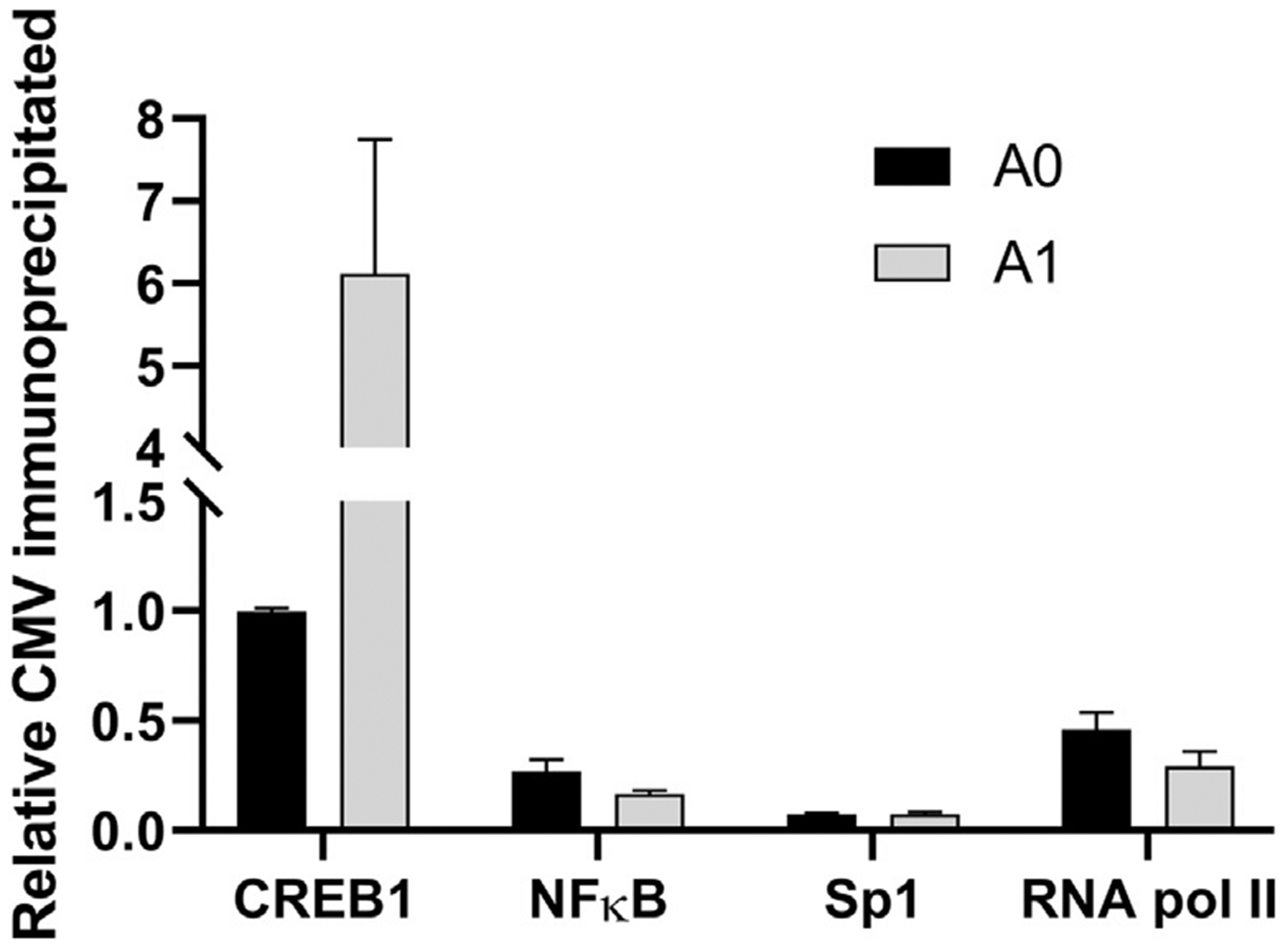
Normalized number of copies of CMV promoter region binding to transcription factors in A1 cell line vs. A0 cell line.

Immunoprecipitated chromatin was purified and then subjected to RT-qPCR to quantify the number of copies of GAPH and CMV promoter regions bound to each transcription factor. After immunoprecipitation, PCR-derived Ct values were normalized to the respective input DNA values to serve as a loading control. We observed a ~6-fold difference in the CREB1 association with the CMV promoter chromatin in the amplified progeny A1 relative to the A0 parental cell line, indicating that the CREB1 association to the CMV promoter was increased even after accounting for MTX amplification and subsequent increase in CMV copies (Fig. 3). The CMV binding to NFκB and RNA pol II was much lower than the binding to CREB1. As expected, the CMV binding to Sp1 was quite low as the Sp1 transcription factor has a predicted low likelihood of association with the CMV promoter.

**Fig. 4.**
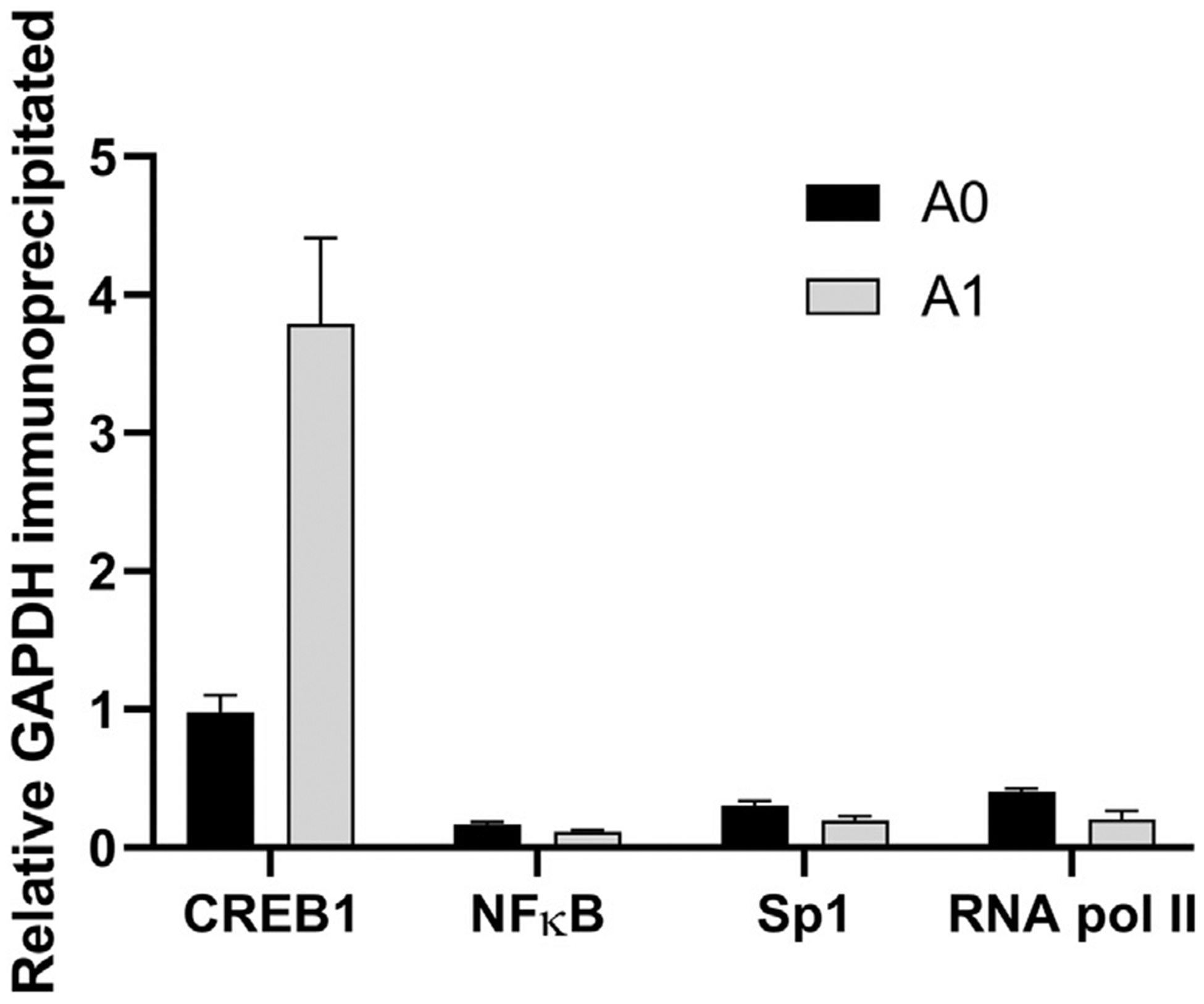
Normalized number of copies of GAPDH promoter region binding to transcription factors in A1 vs. A0 cell line.

When the immunoprecipitated DNA was assayed with GAPDH primer probes (Fig. 4), we observed a ~4-fold increase in the GAPDH-CREB1 association in the amplified clone A1 relative to the progeny clone A0. These data suggest that the transcription factor CREB1 shows an increased association with the genomic DNA within the A1 amplified progeny relative to the parental A0 clone, which would be consistent with activation of CREB1 in the amplified cell lines. Despite the predicted increase in NFκB activity from the URA, no increased association of NFκB or RNA pol II with either CMV or GAPDH was observed.

## 4. Discussion

### 4.1. Role of CREB1 in transcriptional enhancement

In previous studies (Jiang et al., 2006), we observed that the A1 cell line has approximately twice the number of copies of the transgenes but expresses 5-fold more transcripts than the A0 parental clone. Results presented here suggest increased association of CREB1 with the nucleus in the high producer cell line A1 compared with the parental A0 clone. CREB1 is a well-studied transcriptional activator that promotes gene expression from a variety of promoters. CREB1 becomes activated as part of the cAMP cascade. A stimulus from the environment causes cAMP to activate a protein kinase that in turn translocates to the nucleus and phosphorylates the CREB1 protein. Activated CREB1 then binds to its DNA consensus sequence in many promoters in the cell and activates the transcriptional machinery. The consensus sequence (5’-GTGACGT[AC][AG]-3′) is present in many viral and cellular promoters, including the constitutive CMV promoter that is used to drive gene expression in the clones in this study and is widely used for recombinant protein production in the biopharmaceutical industry. (Ortega-Martínez, 2015). Typically, CREB1 binds DNA as a dimer and also acts as a coactivator of transcription by associating with UTF1 (Beausoleil et al., 2004). The CREB-binding protein (CBP) also exhibits histone acetyltransferase (HAT) activity. Hence, CREB1 is suggested to have a role in the activation of transcription by DNA binding as well as modulating the chromatin, specifically by acetylating histones H2B and H4 (Harton et al., 2001).

While CMV is a strong constitutive promoter, it shows great variability in activity depending on the cell line used for transfection (Qin et al., 2010). The CMV promoter also has a large CpG island and is prone to silencing by methylation. Removing CpG islands from the promoter tends to mitigate gene silencing but does not prevent changes in expression due to histone modifications (Ho et al., 2016). While changes in transcription can occur from differences in histone modifications, in the present study we found fairly limited changes in the histone deacetylase (HDAC) and related protein mRNA levels (Supplementary Fig. S1) and even fewer changes at the proteomic and phosphoproteomic levels (Supplementary Table 2) in the clones under study, leading us to focus on other proteomic and phosphoproteomic differences in the amplified clones.

CREB1 is a transcriptional activator that undergoes complex phosphoregulation by multiple kinases at multiple phosphosites. Ser-133 is the most studied phosphosite on CREB1. Phosphorylation of Ser-133 by PKA activates CREB1 by increasing its DNA binding activity, promoting the recruitment of the co-activator proteins CBP and p300. Although we did not find differential phosphorylation of Ser-133 in our phosphoproteomic data, three other phosphopeptides containing four phosphosites were identified as differentially phosphorylated. Various studies have shown phosphosites other than Ser-133 also play a crucial role in CREB1 activity. Hence, it is important to discuss the phosphorylation pattern of these phosphosites to understand the complex mechanism of CREB1 regulation by phosphorylation. For example, we found that phosphopeptide ILNDLsSDAPGVPR (Ser-142) exhibited 1.52-fold higher phosphorylation in the low producer A0 cell line. Phosphorylation of Ser-142 by CamKII inhibits CREB1 transactivation in CV1 mammalian cells by preventing CREB1 dimerization (Wu and McMurray, 2001). Furthermore, the differentially abundant phosphopeptide RLFSGTQISTIAESEDsQEsVDSVTDSQK (Ser-111 and, Ser-114) contains two phosphosites associated with the ataxia-telangiectasia-mutated and casein kinase 1 (ATM/CK) cluster, a group of closely spaced and conserved serine phosphosites at the N-terminal portion of CREB1 (S108, S111, S114, S117 and S121). The phosphorylation of these phosphosites by ATM/CK in response to DNA damage inhibits CREB1 mediated transcription. However, ATM-independent phosphorylation of the ATM/CK cluster positively regulates CREB1-mediated transcription by promoting nuclear translocation of cAMP-regulated transcriptional coactivators (Kim et al., 2016). Also, in response to genotoxic stress, phosphorylation of Ser-111 of CREB1 by ATM primes phosphorylation of Ser-108, Ser-114, and Ser-117 by CK1 and CK2, ultimately leading to inhibitory phosphorylation of Ser-121 on CREB1 by ATM. However, DNA-damage-independent phosphorylation of the ATK/CK cluster does not promote Ser-121 phosphorylation (Shanware et al., 2010). In our data, we did not find phosphorylation of Ser-121, suggesting potential genotoxic stress-independent phosphorylation of the ATM/CK cluster on CREB1 in the high productivity cell line, A1. Genotoxic stress also promotes inhibitory phosphorylation of Ser-271 on CREB1 by homeodomain-interacting protein kinase 2 (HIPK2) (Trinh et al., 2013). We found twofold increased abundance of phosphopeptide TAPTSTIAPGVVMAs SPALPTQPAEEAAR (Ser-271) in the high-producer A1 cell line. Paradoxically, studies have shown an increase in transactivation activity of CREB1 in response to Ser-271 phosphorylation via recruitment of CBP and p300 (Sakamoto et al., 2010). Furthermore, homeodomain interacting protein kinase 2 (HIPK2) has been shown to induce phosphorylation of Ser-2361, Ser-2363, Ser-2371, Ser-2376, and Ser-2381 residues on CBP. It has been suggested, however, that HIPK2 enhances the transcriptional activity of CBP by antagonizing the repressive action of cell cycle regulatory domain 1 (CRD1), located between amino acids 977 and 1076 in HPIK2 independent manner (Kovács et al., 2015). We observed the differential abundance of CBP C-terminal phosphopeptide EEEESSANGTASQstsPSQPR (1061–1083 region) on Ser-1074, Thr-1075 and Ser-1076. The phosphorylation status of these residues has not been investigated for their implication in CBP transactivation activity.

In addition to protein CREB1, the cAMP response element binding protein family also contains transcription factors CREM and ATF, which share a high degree of sequence and structural similarity. As described above, we observed differential abundance of the phosphopeptide containing Ser-271 on CREB1; interestingly, we also found the corresponding peptide of CREM harboring phosphosites Ser-271, Ser-274, Ser-277 and Ser-286 with 1.7-fold higher phosphorylation in high producer A1 cells. Despite being a close member of the CREB family and previously implicated in transcription regulation, CREM and ATF1 transcription factors have not been subjected to the same scientific inquiry as CREB1.

## 5. Concluding remarks

Transcription initiation occurs when the transcriptional machinery binds to the promoter regions of genes. Subsequently, mRNA synthesis occurs and then proteins are trafficked, folded and secreted. Recombinant gene expression in biopharmaceutical processes is frequently driven by the CMV promoter. Many studies have demonstrated the importance of employing vector design, UCOE, and MARs elements to influence the chromatin state to augment the expression of therapeutic proteins in mammalian cells (Veith et al., 2016). However, there is little evidence of the role of the nuclear proteome and the interaction of DNA promoters with transcription factors to demonstrate the mechanism of transcriptional regulation. In this study, two cell clones exhibiting different productivity levels were investigated to determine the mechanism behind higher productivity. From a previous characterization, higher productivity in the selected clones was determined to be influenced by the increased transcription within the higher producer cell lines.

Based upon the binding consensus of the transcription factors in the database, we hypothesized that CREB1 has a large likelihood of binding to the promoter region. By employing ChIP, we were able to study the interactions with the chromatin in each of the cell lines investigated. Our results indicate ~6-fold increased binding in the higher producer cell line. CREB1 is also implicated in other aspects of gene regulation, including histone modifications by CBP, a histone acetyltransferase, (Khan et al., 2017) (Levine et al., 2005) and in interactions with the DNA methyltransferases Dnmt3a and Dnmt3b (Hervouet et al., 2009). As the consensus binding region of the transcriptional factors falls on the site of a CpG island within the CMV promoter, we expect a complex interplay in the function of transcription factor with the DNA in cells adapted to very high productivity phenotypes. Our understanding of the role of CHO nuclear proteome needs to evolve for us to understand the complex interplay between the transcriptional machinery and the high productivity phenotype.

## Supplementary Material

1

2

3

4

## Figures and Tables

**Table 1:** Top scoring transcription factors binding sites. 5 out of 548 sites are shown. From TESS: Transcription Element Search System (http://www.cbil.upenn.edu/cgi-bin/tess/t) along with the *z-score* values from Ingenuity URA analysis.

#	Transcription Factor	Beg	Sns	Len	Sequence	^a^*La*	^b^URA Z-Score
1	T00230 EF-C T00963 MDBP	292	R	25	TATGTTCCCATAGT AACGCCAATAG	50.0	
2	T00029 AP-1 T00590 NF-κB	562	R	23	TTTGACTCACGGGG ATTTCCAAG	46.0	1.74
3	T00051 ATF T00167 CREB1	592	R	18	CCCATTGACGTCA ATGGG	36.0	2.135
4	T00952 AP-2	99	N	8	CCCMNSSS	10.0	

a La: Log likelihood score of association of transcription factor to given consensus sequence.

b URA Z score: Likelihood of activation (positive Z-score) or inactivation (negative Z-score) of transcription factor based on differential expression of genes regulated by the transcription factor.

**Table 2:** CREB1 regulatory relationship proteins from TRRUST (https://www.grnpedia.org/trrust/) transcriptional regulatory database. Fold change values are shown as Log_*2*_FC(A1/A0). Orange cells represent downregulation and green cells represent upregulation in the A1 cell line.

Gene	Activity	RNA-seq	Total Protein	Phosphoprotein	URA-ZSCORE
HMOX1	Repression	−0.70	−1.47	N/A	−2.07
JUN	Repression	−0.42	−1.27	−0.18	−0.34
XPC	Unknown	N/A	−0.76	N/A	N/A
NOLC1	Activation	−0.33	0.89	1.40	N/A
NDC80	Activation	N/A	2.85	N/A	N/A
ETV3	Activation	0.21	N/A	0.61	N/A
