# Supplementary material for: Increased mAb production in amplified CHO cell lines is associated with increased interaction of CREB1 with transgene promoter
Source: Curr Res Biotechnol. Author manuscript; Available in PMC 2020 Jun 23. (PMC7311070; doi:10.1016/j.crbiot.2019.09.001)
Supplement: 1 [file NIHMS1593965-supplement-1.pdf]

Supplementary data

Histone deacetylases (HDACs) are a group of enzymes that remove acetyl groups from lysine residues in histones and non-histone proteins, resulting in transcriptional repression. HDACs have a role in cell growth arrest, differentiation and death. Methylation of the DNA and HDAC activity are closely linked.

We investigated the role of HDACs and related proteins in our cells using RNA extracted from exponentially growing cells. We observed no significant fold changes in the majority of the histone deacetylases or related proteins between in the cell lines. A detailed comparison of the transcriptomic, proteomic and phosphoproteomic changes in HDACs and related proteins is given in Supplementary Table 2.

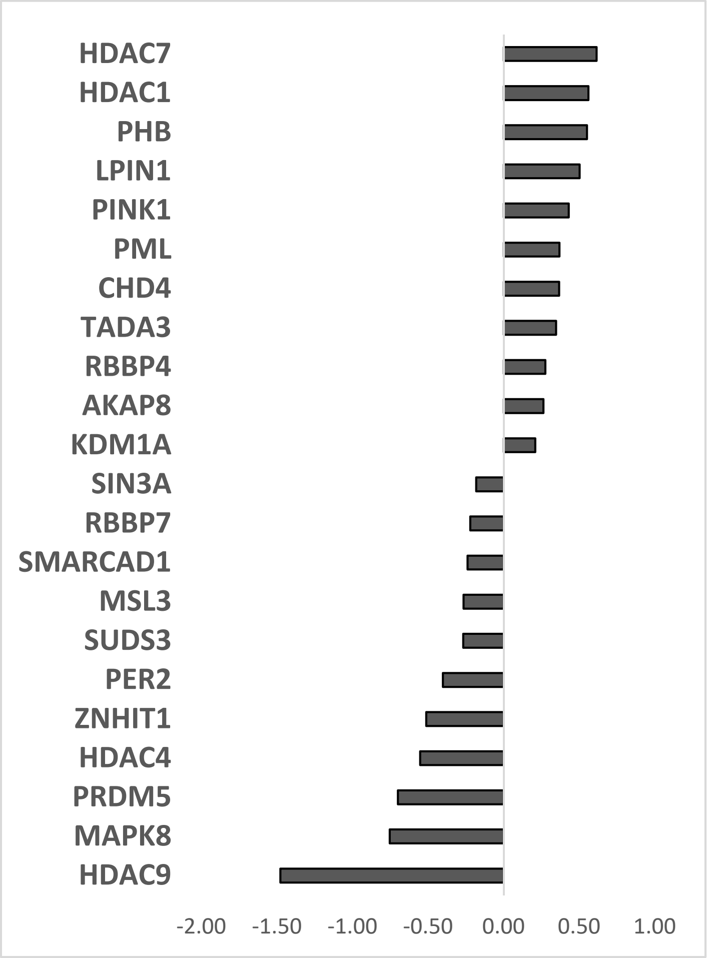

Figure S1: Log_2 Fold-change (A1/A0) of mRNA levels for histone deacetylases and related proteins

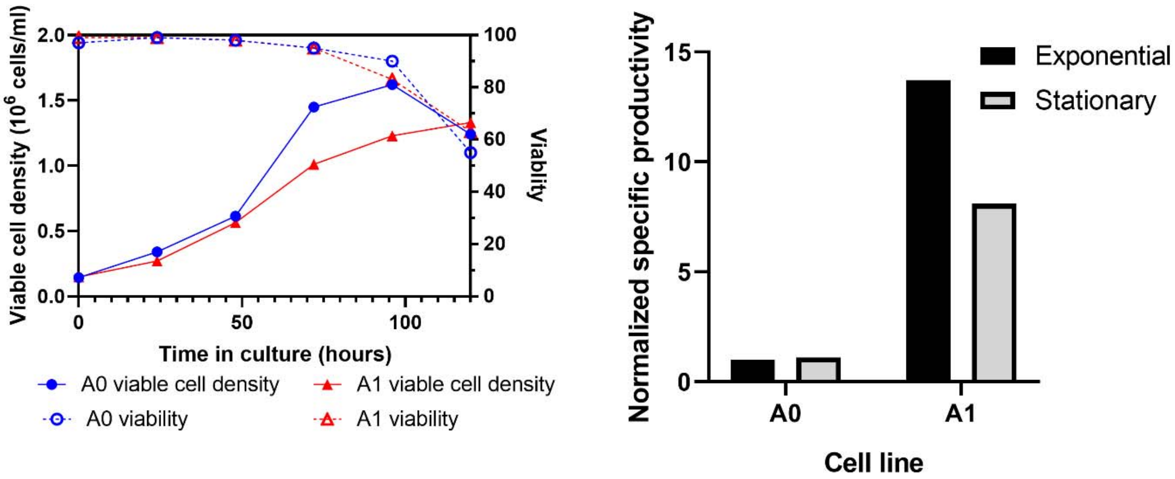

Figure S2: Growth (left panel) and specific productivity (right panel) data for cultures used for RNA-seq and proteomic studies. Samples were taken at 48 hours (exponential) and 96 hours (stationary) for antibody titer measurements. Specific productivities are normalized to the specific productivity for A0 at 48 hours.

Supplementary Table 1 contains complete abundance data for all differentially expressed nuclear proteins, phosphoproteins and phosphopeptides including peptide to spectrum matches. Supplementary Table 1 can be viewed at https://drive.google.com/file/d/1TAZcPS34AhQIs1HXszFbuDWnCFT2WR0Z/view?usp=sharing

Supplementary Table 2 contains differential expression information at the transcriptomic, proteomic and phosphoproteomic levels for histone deacetylases and related proteins.

Supplementary Table 3 contains media composition data.
